# Evaluating the Cassandra NoSQL Database Approach for Genomic Data Persistency

**DOI:** 10.1155/2015/502795

**Published:** 2015-10-19

**Authors:** Rodrigo Aniceto, Rene Xavier, Valeria Guimarães, Fernanda Hondo, Maristela Holanda, Maria Emilia Walter, Sérgio Lifschitz

**Affiliations:** ^1^Computer Science Department, University of Brasilia (UNB), 70910-900 Brasilia, DF, Brazil; ^2^Informatics Department, Pontifical Catholic University of Rio de Janeiro (PUC-Rio), 22451-900 Rio de Janeiro, RJ, Brazil

## Abstract

Rapid advances in high-throughput sequencing techniques have created interesting computational challenges in bioinformatics. One of them refers to management of massive amounts of data generated by automatic sequencers. We need to deal with the persistency of genomic data, particularly storing and analyzing these large-scale processed data. To find an alternative to the frequently considered relational database model becomes a compelling task. Other data models may be more effective when dealing with a very large amount of nonconventional data, especially for writing and retrieving operations. In this paper, we discuss the Cassandra NoSQL database approach for storing genomic data. We perform an analysis of persistency and I/O operations with real data, using the Cassandra database system. We also compare the results obtained with a classical relational database system and another NoSQL database approach, MongoDB.

## 1. Introduction

Advanced hardware and software technologies increase the speed and efficiency with which scientific workflows may be performed. Scientists may execute a given workflow many times, comparing results from these executions and providing greater accuracy in data analysis. However, handling large volumes of data produced by distinct program executions under varied conditions becomes increasingly difficult. These massive amounts of data must be stored and treated in order to support current genomic research [1–4]. Therefore, one of the main problems when working with genomic data refers to the storage and search of these data, requiring many computational resources.

In computational environments with large amounts of possibly unconventional data, NoSQL [5] database systems have emerged as an alternative to traditional Relational Database Management Systems (RDBMS). NoSQL systems are distributed databases built to meet the demands of high scalability and fault tolerance in the management and analysis of massive amounts of data. NoSQL databases are coded in many distinct programming languages and are generally available as open-source software.

The objective of this paper is to study the persistency of genomic data on a particular and widely used NoSQL database system, namely, Cassandra [6]. The tests performed for this study use real genomic data to evaluate insertion and extraction operations into and from the Cassandra database. Considering the large amounts of data in current genome projects, we are particularly concerned with high performances. We discuss and compare our results with a relational system (PostgreSQL) and another NoSQL database system, MongoDB [7].

This paper is organized as follows.  Section 2 presents a brief introduction for NoSQL databases and the main features of Cassandra database system. We discuss some related work in Section 3 and we present, at Section 4, the architecture of the database system.  Section 5 discusses the practical results obtained and Section 6 concludes and suggests future works.

## 2. NoSQL Databases: An Overview

Many relevant innovations in data management came from Web 2.0 applications. However, the techniques and tools available in relational systems may, sometimes, limit their deployment. Therefore, some researchers have decided to develop their own web-scale database solutions [8].

NoSQL (not-only SQL) databases have emerged as a solution to storage scalability issues, parallelism, and management of large volumes of unstructured data. In general, NoSQL systems have the following characteristics [8–10]: (i) they are based on a nonrelational data model; (ii) they rely on distributed processing; (iii) high availability and scalability are main concerns; and (iv) some are schemaless and have the ability to handle both structured and unstructured data.

There are four main categories of NoSQL databases [8, 11–13]:

- Key-value stores: data is stored as key-pairs values. These systems are similar to dictionaries, where data is addressed by a single key. Values are isolated and independent from another, and relationships are handled by the application logic.
- Column family database: it defines the data structure as a predefined set of columns. The* super columns* and* column family *structures can be considered the database schema.
- Document-based storage: a document store uses the concept of key-value store. The documents are collections of attributes and values, where an attribute can be multivalued. Each document contains an ID key, which is unique within a collection and identifies document.
- Graph databases: graphs are used to represent schemas. A graph database works with three abstractions: node, relationships between nodes, and key-value pairs that can attach to nodes and relationships.

### 2.1. Cassandra Database System

Cassandra is a cloud-oriented database system, massively scalable, designed to store a large amount of data from multiple servers, while providing high availability and consistent data [6]. It is based on the architecture of Amazon's Dynamo [14] and also on Google's BigTable data model [15]. Cassandra enables queries as in a key-value model, where each row has a unique row key, a feature adopted from Dynamo [6, 14, 16, 17]. Cassandra is considered a hybrid NoSQL database, using characteristics of both key-value and column oriented databases.

Cassandra's architecture is made of nodes, clusters, data centers and a* partitioner*. A node is a physical instance of Cassandra. Cassandra does not use a master-slave architecture; rather, Cassandra uses peer-to-peer architecture, which all nodes are equal. A cluster is a group of nodes or even a single node. A group of clusters is a data center. A* partitioner* is a hash function for computing the token of each row key.

When one row is inserted, a token is calculated, based on its unique row key. This token determines in what node that particular row will be stored. Each node of a cluster is responsible for a range of data based on a token. When the row is inserted and its token is calculated, this row is stored on a node responsible for this token. The advantage here is that multiple rows can be written in parallel into the database, as each node is responsible for its own write requests. However this may be seen as a drawback regarding data extraction, becoming a bottleneck. The* MurMur3Partitioner* [17] is a partitioner that uses tokens to assign equal portions of data to each node. This technique was selected because it provides fast hashing, and its hash function helps to evenly distribute data to all the nodes of a cluster.

The main elements of Cassandra are* keyspaces*, column families, columns, and rows [18]. A* keyspace* contains the processing steps of the data replication and is similar to a schema in a relational database. Typically, a cluster has one* keyspace* per application. A column family is a set of key-value pairs containing a column with its unique row keys. A column is the smallest increment of data, which contains a name, a value, and a timestamp. Rows are columns with the same primary key.

When a write operation occurs, Cassandra immediately stores the instruction on the Commit log, which goes into the hard disk (HD). Data from this write operation is stored at the* memtable*, which stays in RAM. Only when a predefined memory limit is reached, this data is written on* SSTables* that stay in the HD. Then, the Commit log and the* memtable* are cleaned up [18, 19]. In case of failure regarding the* memtables*, Cassandra reexecutes the written instructions available at the Commit log [19, 20].

When an extract instruction is executed, Cassandra first searches information in* memtables*. A large RAM allows large amounts of data in* memtables* and less data in HD, resulting in quick access to information [16].

## 3. Storing Genomic Data

Persistency of genomic data is not a recent problem. In 2004, Bloom and Sharpe [21] described the difficulties of managing these data. One of the main difficulties was the growing number of data generated by the queries. The work in Röhm and Blakeley [22] and Huacarpuma [23] consider relational databases (SQL Server 2008 and PostgreSQL, resp.) to store genomic data in FASTQ format.

Bateman and Wood [24] have suggested using NoSQL databases as a good alternative to persisting genetic data. However, no practical results are given. Ye and Li [25] proposed the use of Cassandra as a storage system. They consider multiple nodes so that there were no gaps in the consistency of the data. Wang and Tang [26] indicated some instructions for creating an application to perform data operations in Cassandra.

Tudorica and Bucur [27] compared some NoSQL databases to a MySQL relational database using the YCSB (Yahoo! Cloud Serving Benchmark). They conclude that in an environment where write operations prevail MySQL has a significantly higher latency when compared to Cassandra. Similar results about performance improvements for writing operations in Cassandra, when compared to MS SQL Express, were also reported by Li and Manoharan [28].

Many research works [25–28] present results involving the performance of a Cassandra database system for massive data volumes. In this paper, we have decided to evaluate the performance of Cassandra NoSQL database system specifically for genomic data.

## 4. Case Study

To validate our case study we have used real data. The sequences (also called* reads*) were obtained from liver and kidney tissue samples of one human male from the SRA-NCBI (http://trace.ncbi.nlm.nih.gov/Traces/sra/sra.cgi?), sequenced by the Illumina Genome Analyzer. It produced 72,987,691 sequences for the kidney samples and 72,126,823 sequences for the liver samples, each sequence containing 36 bases. Marioni et al. [29] generated these sequences.

FASTQ file stores sequences of nucleotides and their corresponding quality values. Three files were obtained from filtered sequences sampled from kidney cells, and another three files consisted of filtered genomic sequences sampled from liver cells. It should be noted that these data were selected because they were in FASTQ [1] format, which is commonly used in bioinformatics workflows.

In this case study, we carried out three analyses. In the first one, we investigated how Cassandra behaves when the computational environment is composed of a cluster with two and four computers. In the second one, we analyze the behavior of Cassandra compared to PostgreSQL, a relational database. In the last case study, we used the MongoDB document-oriented NoSQL database to compare to Cassandra's results.

### 4.1. Cloud Environment Architecture

In order to investigate the expected advantages of Cassandra's scalability, we have created two cloud environments: one with two nodes and the other with four nodes. Cassandra was installed on every node of the cluster. We have also used* OpsCenter* 4.0 [30], a DSE tool that implements a browser-based interface to remotely manage the cluster configuration and architecture. The architecture contains a single data center, named DC1. A single cluster, named* BIOCluster*, containing the nodes, was created, working with DC1.

### 4.2. Java Client

At the software level, we have defined the following functional requirements: (i) create a keyspace; (ii) create a table to store a FASTQ file; (iii) create a table with the names of inserted FASTQ files and their corresponding metadata; (iv) receive an input file containing data from a FASTQ file and insert it into a previously created table, followed by the file name and metadata; (v) extract all data from a table containing the contents of a FASTQ file; and (vi) remove the table and the keyspace.

Nonfunctional requirements were also defined: (i) the use of Java API, provided by DataStax, in order to have a better integration between the Cassandra distribution and the developed client application; (ii) the use of Cassandra Query Language (CQL3) [17], for database interactions, which is the current query language of Cassandra and resembles SQL; (iii) conversion to JSON files to be used by the client application, since it is simpler to work with JSON files in Java; and (iv) a good performance in operations.

With respect to this last requirement, three applications were developed, two for data conversion and one client application for Cassandra.

**Figure 1:**
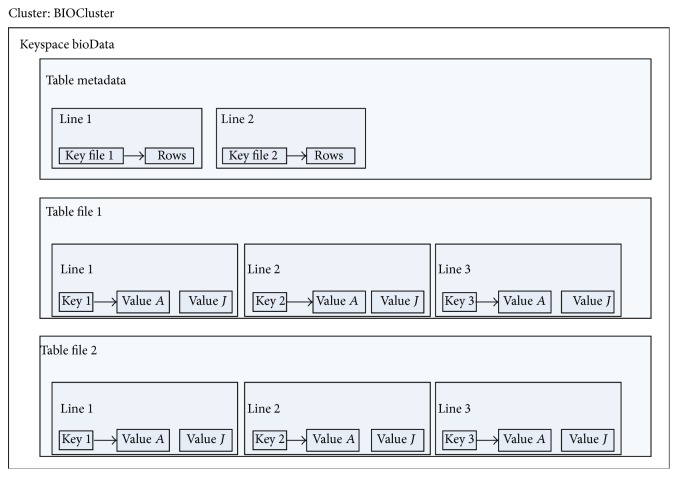
Database schema.

**Figure 2:**
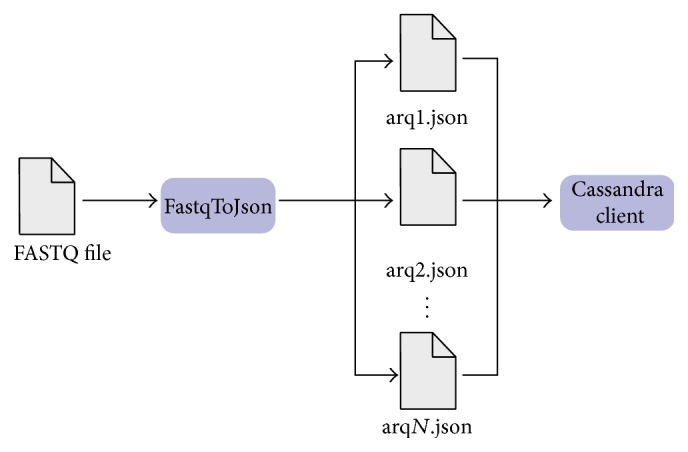
Stages of insertion.

**Figure 3:**
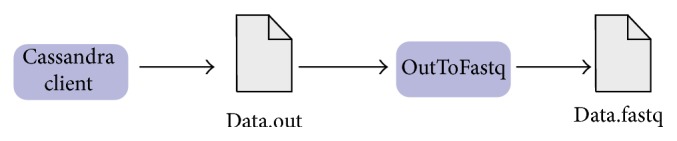
Stages of extraction.

1. FastqTojson Application converts the FASTQ input file into smaller JSON files, each JSON file with five hundred thousand reads. The objective is to load these small JSON files because, usually, FASTQ file occupies a few gigabytes. Furthermore, as it presents a proper format for the Java client, it does not consume many computational resources. Each JSON file occupies ten thousand rows in the database: each row is an array of ten columns; each field value of the column contains five* reads*.
2. Cassandra client was also developed in Java, using the Java API provided by DataStax and is the one in which the data persists. This client creates a keyspace, inserts all JSON files from the first application in a single table, and extracts the data from a table.
3. For the database schema, it consists of a single* keyspace*, called* biodata*, a single cluster, called* biocluster*, one table of metadata and one table for each file persisting, as shown in Figure 1.
4. The allocation strategy for replicas and the replication factor are properties from the* keyspace*. The allocation strategy determines whether or not data is distributed through a network of different clusters. The Simple Strategy [31] was selected since this case study was performed in a single cluster. Likewise, since we did not consider failures and our goal was to study performance rather than fault recovery, we have chosen one replication factor. It should be noted that the replication factor determines the number of replicas distributed along the cluster. Focusing on performance, a higher number of replicas would also interfere on the insertion time.
5. As previously mentioned, the client application creates a table for each inserted FASTQ file, which has the same name of the file. Each of these tables has eleven columns, and each cell stores a small part of a JSON file, ten* reads* per cell, which is about 1 MB in size. This small set for columns and cells is due to the efficiency of Cassandra when a small number of columns are used and a big number of rows. This is also a consequence of the ability of* MurMur3Partitioner* to distribute each row in one node. Therefore, the cluster has a better load balance during insertions and extractions.
6. Once a table is created, the client inserts all data from JSON in the first stage on the database, as shown in Figure 2. In what follows, a single row is inserted into the metadata table containing as a row key the name of FASTQ file and a column with the number of rows. This latter is inserted into the metadata table to solve the memory limit of the Java Virtual Machine, which may happen when querying large tables.
7. When extracting data, the client queries the metadata table to get the number of rows on the table with the FASTQ data and then proceeds to the table extraction, which is done row by row and written into an “.out” file.
8. OutToJso Application. After data extraction, there is a single file with the extension “.out.” This application converts this file into a FASTQ format, making it identical to the original input file, resulting only in the FASTQ file without temporary file “.out.” This process is shown in Figure 3.

## 5. Results

In this work, we have considered three experimental case studies to evaluate data consistency and performance for storing and extracting genomic data. For the first one, we verified Cassandra's scalability and variation in performance. For the second case study, we compared the Cassandra results to a PostgreSQL relational system and, finally, we used the MongoDB NoSQL database and compared other results to Cassandra NoSQL system. The case studies used the same data to insert and read sequences.

During the Cassandra evaluation, we have created two clusters. The first one, a Cassandra cluster with two computers, was created, while for the second one, a new cluster with four computers was created. The first cluster consisted of two computers with Intel Xeon E3-1220/3.1 GHz processor, one with 8 GB RAM and the other with 6 GB RAM. For the second cluster, besides the same two computers, two other computers with Intel Core i7 processor and 4 GB RAM was included. Each one of them used Ubuntu 12.04.

### 5.1. Insertions and Extractions Cassandra NoSQL

The input files are six FASTQ files with filtered data from kidney and liver cells.  Table 1 shows the sizes of the file and the number of rows that their respective JSON file had when inserted into Cassandra.

We have based the performance analyses on the elapsed time to store (insert) data into and to retrieve (extract) data from the database. These elapsed times are important because if one wants to use the Cassandra system in bioinformatics workflows, it is necessary to know how long the data becomes available to execute each program.

Table 2 shows the elapsed times to insert and extract sequences in the database, with both implementations. Columns 3 and 5 show the insertions using two nodes. Similarly, columns 4 and 6 show the extractions using four nodes. As expected, we could confirm the hypothesis that the database performance increases when we add more nodes.

**Figure 4:**
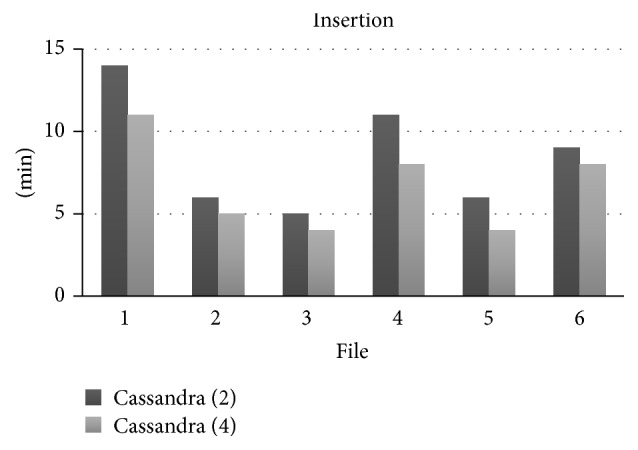
Comparison between inserts (time × file number).

**Figure 5:**
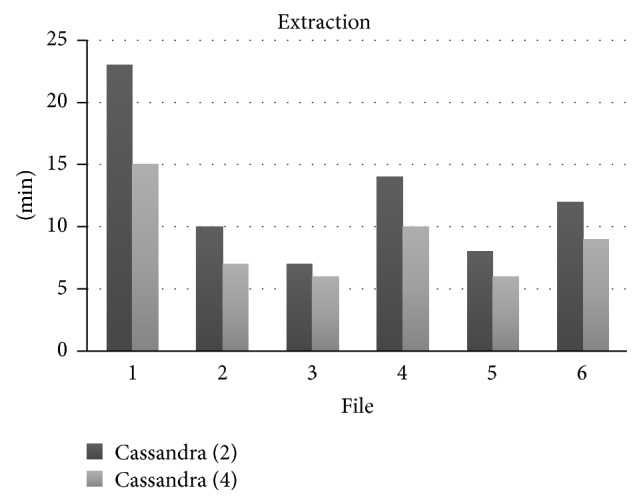
Comparison between extractions (time × file number).

Figures 4 and 5 show comparative charts of insertion and extraction elapsed times according to the number of computers that Cassandra considers. Insertion into two computers is longer than using four computers. Here the performance also improves when the number of computers increases in the cluster.

### 5.2. Comparison of Relational and Cassandra NoSQL Systems

We compared the Cassandra results with Huacarpuma [23] that used the same data to insert and read sequences in the PostgreSQL, a relational database. In the latter experiment, the author used only one server with an Intel Xeon processor, eight cores of 2.13 GHz and 32 GB RAM, executing Linux Server Ubuntu/Linaro 4.4.4-14.

The server's RAM for the relational database is larger than the sum of the memories of the four computers used in this experiment. Nonetheless, we use the results of the relational database to demonstrate that it is possible to achieve high performances even with a modest hardware due to scalability and parallelism.

Table 3 shows the sum of the insertion and extraction times in the relational database and the two computational environments using Cassandra, Cassandra (2), a cluster with two computers, and Cassandra (4), a cluster with four computers.

**Figure 6:**
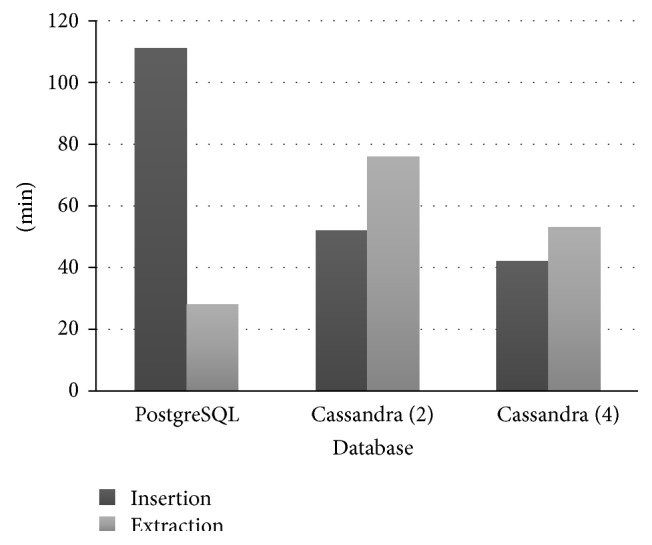
Comparison between Cassandra and PostgreSQL.

The writing time in Cassandra is lower due to parallelism, as seen in Table 3. Write actions in Cassandra are more effective than in a relational database. However, its performance was lower for query answering, as shown in Figure 6. This is due to two factors: first, Cassandra had to ensure that the returned content was in its latest version, verifying the data divided between machines; second, the data size is larger than the available RAM; therefore, part of the data had to be stored in SSTable, reducing the speed of the search.

The reader should note that the results obtained with Cassandra just indicate a trend. They are not conclusive because the hardware characteristics of all experiments are different.

Nevertheless, the improved performance with the increase of nodes is an indication that Cassandra may sometimes surpass relational database systems in a larger number of computers, making its use viable in data searches in bioinformatics.

### 5.3. Comparison of MongoDB and Cassandra NoSQL Databases

We compared the Cassandra results to the same data to insert and read sequences in a MongoDB NoSQL. This is an open-source document-oriented NoSQL database designed to store large amounts of data.

The server where we have installed MongoDB is an i7 processor with 16 GB RAM. This server has 2 GB RAM more. The server where we have installed MongoDB had 2 GB RAM more than cluster with two computers, Cassandra (2), and 6 GB RAM less than the sum of the RAM memories of four computers, Cassandra (4).

Table 4 shows the sum of the insertion and extraction times in the MongoDB database and the Cassandra with two and four computers in a cluster. The performances of insertion operations were similar using either MongoDB or Cassandra databases. However, the MongoDB showed better behavior than Cassandra NoSQL in the extraction of genomic data in FASTQ format.

**Figure 7:**
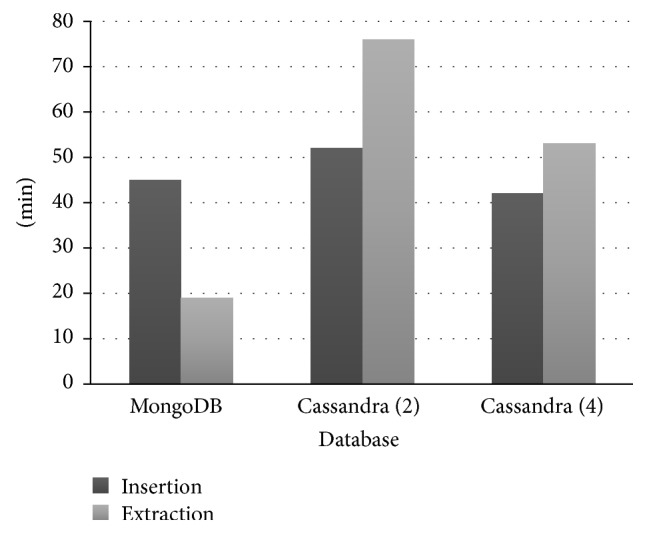
Comparison between Cassandra and MongoDB database.

In Figure 7 our results suggest that there is a similar behavior of the insertions in both MongoDB and Cassandra. There was a performance gain of more than 50% in the extraction, when comparing the results of a Cassandra in a cluster with two computers and another cluster with four computers.

## 6. Conclusions

In this work we studied genomic data persistence, with the implementation of a NoSQL database using Cassandra. We have observed that it presented a high performance for writing operations due to the larger number of massive insertions compared to data extractions. We used the DSE tool together with Cassandra, which allowed us to create a cluster and a client application suitable for the expected data manipulation.

Our results suggest that there is a reduction of the insertion and query times when more nodes are added in Cassandra. There was a performance gain of about 17% in the insertions and a gain of 25% in reading, when comparing the results of a cluster with two computers and another cluster with four computers.

Comparing the performance of Cassandra to the MongoDB database, the results of MongoDB indicate that the extraction of the MongoDB is better than Cassandra. For data insertions the behaviors of Cassandra and MongoDB were similar.

From the results presented here, it is possible to outline new approaches in studies of persistency regarding genomic data. Positive results could boost new research, for example, the creation of a similar application using other NoSQL databases or new tests using Cassandra with different hardware configurations seeking improvements in performance. It is also possible to create a relational database with hardware settings identical to Cassandra, in order to make more detailed comparisons.

## Figures and Tables

**Table 1:** Cells files.

File	File number	Size	Number of lines
Liver cells files	1	9,0 GB	850.933
	2	4,0 GB	358.841
	3	3,2 GB	286.563

Kidney cells files	4	6,9 GB	648.612
	5	3,8 GB	335.973
	6	5,3 GB	475.210

**Table 2:** Times to insert and extract sequences from the database.

File	Size	Insertion		Extraction	
		Cassandra (2)	Cassandra (4)	Cassandra (2)	Cassandra (4)
1	9,0 GB	14 m 30 s 645 ms	11 m 44 s 105 ms	23 m 37 s 964 ms	15 m 04 s 158 ms
2	4,0 GB	6 m 10 s 471 ms	05 m 05 s 710 ms	9 m 41 s 018 ms	7 m 34 s 523 ms
3	3,2 GB	5 m 05 s 914 ms	4 m 51 s 823 ms	7 m 39 s 188 ms	6 m 02 s 648 ms
4	6,9 GB	11 m 25 s 899 ms	8 m 27 s 630 ms	14 m 25 s 120 ms	10 m 00 s 031 ms
5	3,8 GB	6 m 09 s 417 ms	4 m 42 s 386 ms	8 m 37 s 890 ms	6 m 05 s 487 ms
6	5,3 GB	8 m 43 s 330 ms	8 m 05 s 215 ms	12 m 23 s 855 ms	9 m 03 s 041 ms

**Table 3:** PostgreSQL and Cassandra results.

Database	Insertion	Extraction
PostgreSQL	1 h 51 m 54 s	28 m 27 s
Cassandra (2)	52 m 5 s	1 h 16 m 25 s
Cassandra (4)	42 m 56 s	53 m 49 s

**Table 4:** MongoDB and Cassandra final results.

Database	Insertion	Extraction
MongoDB	45 m 17 s	19 m 13 s
Cassandra (2)	52 m 5 s	1 h 16 m 25 s
Cassandra (4)	42 m 56 s	53 m 49 s
